# Supplementary material for: Bibliometric Analysis: Six Decades of Scientific Production from a Nationwide Institution: Instituto de Seguridad y Servicios Sociales de los Trabajadores del Estado (ISSSTE) from Mexico
Source: Healthcare (Basel). 2023 Jun 12;11(12):1725. doi: 10.3390/healthcare11121725 (PMC10298242; doi:10.3390/healthcare11121725)
Supplement: Supplementary file 1 [file healthcare-11-01725-s001.zip › healthcare-2294423-supplementary.pdf]

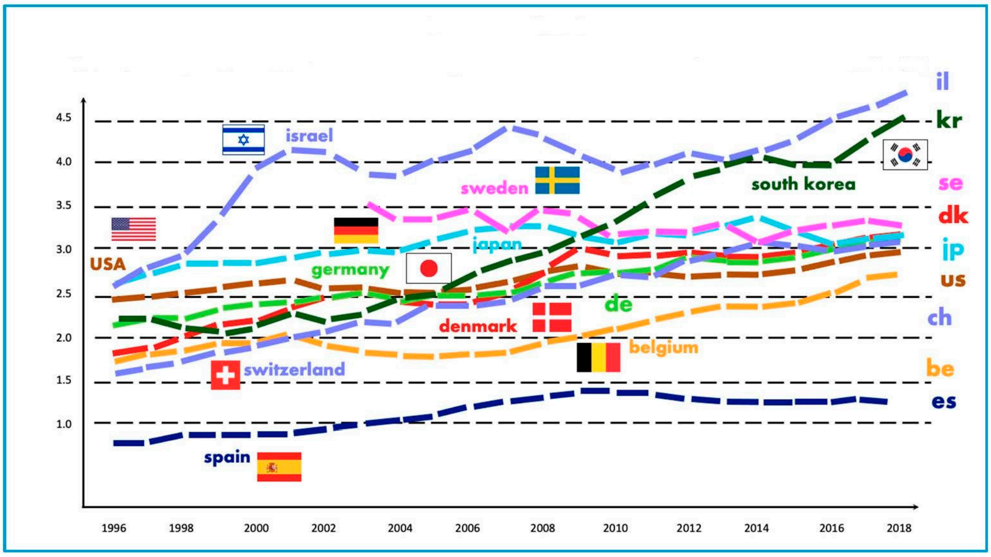

Figure S1: Representation (World Bank Data) showing the percentage investment in science and R&D of developed countries (1996–2020).
